# Chronic wasting disease (CWD) prion detection in blood from pre-symptomatic white-tailed deer harboring *PRNP* polymorphic variants

**DOI:** 10.1038/s41598-020-75681-7

**Published:** 2020-11-13

**Authors:** Carlos Kramm, Paulina Soto, Tracy A. Nichols, Rodrigo Morales

**Affiliations:** 1 Department of Neurology, McGovern Medical School, The University of Texas Health Science Center at Houston, Houston, TX 77030 USA; 2 Facultad de Medicina, Universidad de Los Andes, Las Condes, Av. San Carlos de Apoquindo 2200, Santiago, Chile; 3 United States Department of Agriculture, Animal Plant Health Inspection Service, Veterinary Services, Fort Collins, CO 80526 USA; 4 CIBQA, Universidad Bernardo O’Higgins, Santiago, Chile

**Keywords:** Prion diseases, Prions, Biochemistry, Neuroscience

## Abstract

Chronic wasting disease (CWD) is a prionopathy affecting wild and farmed cervids. This disease is endemic in North America and has been recently identified in Europe. Ante-mortem CWD tests of pre-clinical cervids may be an important tool in helping control the spread of this disease. Unfortunately, current CWD diagnostic methods are not suitable for non-tissue type samples. We reported that CWD prions can be detected in blood of pre-clinical CWD-infected white-tailed deer (WTD) with high sensitivity and specificity using the Protein Misfolding Cyclic Amplification (PMCA) assay. However, that report only included animals homozygous for codon 96G, the most common polymorphic version of the prion protein within this animal species. Here, we report CWD prion detection using blood of naturally infected WTD coding one or two copies of the PrP-96S polymorphic variant. Our results, from a blinded screening, show 100% specificity and ~ 58% sensitivity for animals harboring one 96S codon, regardless of their stage within the pre-clinical phase. Detection efficiency for PrP-96S homozygous animals was substantially lower, suggesting that this allele affect peripheral prion replication/tropism. These results provide additional information on the influence of codon 96 polymorphisms and the ability of PMCA to detect CWD in the blood of pre-clinical WTD.

## Introduction

Chronic wasting disease (CWD) is the only prion disease found in free-ranging animals^1–3^. Screening and containment programs aimed at halting CWD spread have been established with variable success^4–6^. Unfortunately, the lack of practical diagnostic methods has been one of the factors stopping the implementation of more efficient containment practices. At present, the gold-standard method for CWD diagnosis is the immunohistochemical (IHC) analysis of disease-associated prion protein (PrP^Sc^) accumulation in medial retropharyngeal lymph nodes and obex (https://www.aphis.usda.gov/animal_health/animal_diseases/cwd/downloads/cwd-program-standards.pdf). The fact that several bodily fluids and excreta from CWD infected animals carry prion infectivity^7–9^ provides potential targets for animal diagnostic testing. Our lab and others have shown that in vitro prion replication techniques (such as the protein misfolding cyclic amplification (PMCA)^10^ and real-time quaking induced conversion (RT-QuIC)^11^) have the potential for use in pre-clinical diagnosis using a wide variety of biological samples. In a previous publication^12^, we communicated the results of CWD prion screening using blood from a group of farm raised pre-symptomatic white-tailed deer (WTD). Our results showed 96% sensitivity at late pre-symptomatic stages, and 53% at early stages. The specificity of our assay was 100%. These results suggested that PMCA can identify a fraction of CWD positive animals.

A drawback from the study mentioned above is that data was collected using specimens from animals harboring the most common prion protein (PrP^C^) sequence only. PrP^C^ polymorphisms are seen in several animal species, including cervids^2,13–16^. In WTD, the most commonly found polymorphism occurs at position 96 where a glycine (G) is substituted for a serine (S)^17^. The presence of at least one S at position 96 is associated with longer incubation periods compared to animals expressing the most common (“wild-type”, PrP 96GG) genotype^17,18^. Importantly, animals carrying PrP 96S allele(s) have been associated with lower prion shedding, suggesting lower accumulation of PrP^Sc^ in peripheral tissues compared to their wild-type counterpart^19,20^. This variable could have important implications for pre-clinical diagnosis when using peripheral tissue biopsies, bodily fluids and excreta. In this work, we evaluated the diagnostic efficiency of the PMCA technology using blood specimens collected from farmed/non-symptomatic WTD carrying one or two PrP 96S allele(s).

## Materials and methods

### Ethics statement

Blood samples from male and female WTD were collected from farmed herds depopulated due to CWD by the USDA. These samples were sent to Dr. Morales’ group for prion screening using the PMCA technology. The investigators at Dr. Morales’ lab were blinded to the identity of the samples when performing the PMCA assay. All work manipulating these samples was previously approved by the Institutional Biosafety Committee of The University of Texas Health Science Center at Houston (UTHealth). PMCA substrate was derived from brains of transgenic mice expressing the WTD prion protein expressing glycine at position 96 (Tg1536^21^). Procedures for animal handling were approved by the Institutional Animal Care and Use Committee at UTHealth.

### Study populations

The animal population included subjects coding for the 96S polymorphic variant of the prion protein (81% heterozygous, 19% homozygous). All animals were devoid of classical CWD clinical signs (including but not limited to neurological abnormalities, polydipsia, polyuria, weight loss, etc.) as assessed by Dr. Tracy Nichols and/or other qualified USDA personnel. These animals were sacrificed immediately after blood collection. CWD status was assessed by the presence of protease-resistant PrP aggregates by IHC in the obex and retropharyngeal lymph nodes (RPLN). CWD staging was assigned as follows: obex (+) and RPLN (+): late pre-symptomatic; obex (−) and RPLN (+): early pre-symptomatic; obex (−) and RPLN (−): non-detect. In summary, blood samples from 42 animals (12 late pre-symptomatic, 19 early pre-symptomatic, and 11 CWD-non detect) were used in this study. The specific data for blood donors is displayed in Table 1.

**Table 1:** Individual data of white tail deer used in this study.

Animal ID	CWD status by IHC*	PrP (at position 96)	PMCA result	Animal ID	CWD status by IHC*	PrP (at position 96)	PMCA result
037	Obex/RPLN	GS	+	163	RPLN	GS	+
053	Obex/RPLN	GS	+	181	RPLN	GS	+
063	Obex/RPLN	GS	−	184	RPLN	GS	+
064	Obex/RPLN	GS	−	217	RPLN	GS	−
065	Obex/RPLN	GS	+	287	RPLN	GS	−
077	Obex/RPLN	GS	+	029	nd	GS	−
111	Obex/RPLN	GS	−	043	nd	GS	−
123	Obex/RPLN	GS	−	054	nd	GS	−
129	Obex/RPLN	GS	+	074	nd	GS	−
134	Obex/RPLN	GS	+	083	nd	GS	−
142	Obex/RPLN	GS	−	091	nd	GS	−
166	Obex/RPLN	GS	+	093	nd	GS	−
040	RPLN	GS	+	270	nd	GS	−
042	RPLN	GS	−	085	RPLN	SS	−
080	RPLN	GS	−	094	RPLN	SS	−
087	RPLN	GS	−	116	RPLN	SS	−
092	RPLN	GS	−	159	RPLN	SS	+
108	RPLN	GS	+	160	RPLN	SS	−
113	RPLN	GS	−	089	nd	SS	−
133	RPLN	GS	−	100	nd	SS	−
140	RPLN	GS	+	109	nd	SS	−

*Indicates positive detection of PK resistant PrP in Obex or medial retropharyngeal lymph node (RPLN) tissues by immunohistochemistry (nd: non detected).

### Blood sample collection

Blood was collected from the jugular vein of chemically immobilized animals using commercially available blood collection tubes containing EDTA as anticoagulant. Specimens were frozen and stored at − 80 °C at USDA facilities (Fort Collins, CO). These samples were overnight-transferred to UTHealth in containers filled with ice packs. Once at UTHealth, samples were stored again at − 80 °C until use.

### Sample processing

Blood specimens were thawed at room temperature and vortexed. One aliquot (10 µL) of each of these samples was directly mixed with PMCA substrate, while a second aliquot (200 µL) was processed for PrP^Sc^ concentration by ultracentrifugation as previously described^10,12,22^. Briefly, the 200 µL blood aliquots were mixed with equivalent volumes of a 20% w/v sarkosyl solution (prepared in PBS) for 1 h at room temperature and then centrifuged at 100,000×*g* for 1 h at 4 °C. Supernatants were carefully discarded without disturbing pellets, 400 µL of PBS added to them, and centrifuged using the same protocol described above but for just 30 min. Resulting pellets were resuspended in PMCA conversion buffer and transferred to 200 µL PCR tubes.

### PMCA assay and western blotting

**Figure 1:**
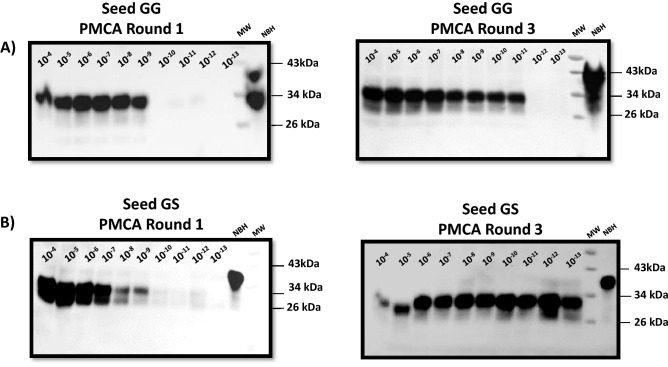
In vitro prion replication of WTD brain-derived PrP 96GG and PrP 96GS CWD prions. Serial dilutions of normalized PK-resistant PrP 96GG (**A**) and PrP 96GS (**B**) CWD prions (brain-derived) were loaded into PMCA reactions and tested for their replication efficiencies. This figure depict results of the 1st (left panels) and 3rd (right panels) PMCA rounds. MW represent molecular weight protein standards. Normal Brain Homogenate (NBH) represents non PK-treated brain extracts from Tg1536 mice used as molecular weight migration and antibody specificity controls. Numbers at the right of right panels represent molecular weight markers (in KDa).

**Figure 2:**
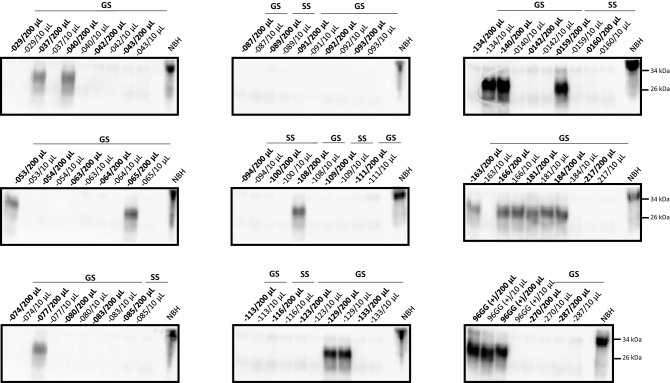
CWD prion detection in blood samples from WTD expressing for PrP 96S. PK-treated PMCA products from WTD-derived blood are depicted. Blood samples were collected from WTD expressing for at least one copy of the PrP 96S polymorphic variant. Blood samples were tested in PMCA by either direct spiking (10 µL) or concentration (200 µL) as described in Materials and Methods. “GS” or “SS” at the top of each panel depict PrP polymorphic classification. Sample ID is represented in each lane using the “-XX” format. 96GG (+) represent blood of late pre-symptomatic PrP 96GG deer used as positive control. Normal Brain Homogenate (NBH) represents non PK-treated brain extracts from Tg1536 mice used as molecular weight migration and antibody specificity controls. Numbers at the right of right panels represent molecular weight markers (in KDa). This figure depict two of the four replicates tested for each sample. Samples − 080 and − 092 were PMCA positive in a different set of replicates (not shown).

The PMCA procedure was performed as described previously^10,12,23^. Briefly, each blood sample was run in quadruplicates considering the modalities explained above: direct spiking and concentration (samples run twice for each modality). PMCA products were treated with 100 µg/mL proteinase K (PK) at 37 °C and constant shaking for 1 h. PK reaction was stopped by adding LDS sample buffer and exposure to 95 °C for 10 min. Later, samples were fractionated by SDS-PAGE and transferred to nitrocellulose membranes as described^10^. Membranes were probed with either 6H4 (Fig. 1) or PRC1 (Fig. 2) antibodies at 1:10,000 or 1:5000 dilutions, respectively. Membranes were developed using a chemoluminiscence method as explained in previous publications^10,22,24^. A blood sample was considered as CWD positive if at least one of the replicates exhibited PK resistant PMCA products, as previously described^12^. Readouts were taken at the 4th PMCA round.

### Statistical analysis

Overall correct diagnosis was calculated using the following formula: (correct PMCA diagnosis in both positive and negative groups/total number of cases) × 100. Estimated predictive values for sensitivity and specificity were calculated using the MedCalc software (https://www.medcalc.org/calc/diagnostic_test.php).

### Ethical approval

The experiments listed in this manuscript did not involved animal experimentations. Blood samples from WTD were collected previously for other purposes by TN. We have USDA approval to receive and work with these samples. As substrate for PMCA, we used brains from transgenic mice expressing deer PrP^C^. The procedures for breeding, manipulations and euthanasia of these transgenic mice was performed following NIH guidelines and approved by the Animal Welfare Committee of The University of Texas Health Science Center at Houston (protocol AWC-17-0049).

### Original western blot files

Original files for the figures included in this article can be found at Supplemental Figure S1.

## Results

Several reports involving diverse animal species suggest that prion transmission may be influenced by inter-polymorphic prion factors^25^. For that reason, we first evaluated the efficiency of our previously published cervid PMCA system^12,23^ that utilized substrate homozygous for Glycine at codon 96 in the *PRNP* gene. This experiment was done as this substrate is not homologous to the *PRNP* polymorphisms of the WTD samples used in this study. Specifically, brain extracts from experimentally infected PrP 96GG and PrP 96GS WTD (kindly provided by Dr. Edward Hoover) were used to compare in vitro amplification rates. After normalizing the amount of PK-resistant PrP in both inocula by western blot, serial dilutions in PMCA substrate were performed. The PMCA reaction using the PrP 96GG inoculum provided amplification up to 1 × 10^−6^–1 × 10^−7^ dilutions in the first PMCA round. At the third PMCA round, positive prion replication was reproducibly observed up to 1 × 10^−11^ dilution for this particular inoculum (Fig. 1A). For the PrP 96GS sample, a similar amplification compared to its PrP 96GG counterpart was observed in the first PMCA round. At PMCA round three, we observed a higher amplification as PK-resistance PrP signals were observed at the 1 × 10^−13^ dilution (Fig. 1B). This data shows that our current PMCA system is suitable to screen samples carrying at least one PrP 96S allele. Unfortunately, a similar experiment using PrP 96SS CWD prions was not possible since we did not have access to this sample type.

Considering these results, we analyzed blood samples from CWD-infected WTD harboring at least one PrP 96S allele using the PMCA system described in Fig. 1. As detailed in Materials and Methods and Table 1, we analyzed a total of 42 blood samples collected from 31 CWD positive and 11 CWD non-detect animals with either the GS or SS genotype at *PRNP* codon 96. Prion infected subjects were distributed in 12 (39%) late pre-symptomatic and 19 (61%) early pre-symptomatic cases (Table 2). Readings taken at the 4th PMCA round confirmed the specificity of our assay as no samples from the CWD-non detect animals provided PK-resistant PrP signals (Table 2, Fig. 2). In blood samples from PrP 96GS animals, we observed 57.1% (8/14) detection accuracy in the early pre-symptomatic CWD stage and a similar detection accuracy of 58.3% (7/12) in the late pre-symptomatic stage. For the PrP 96SS group, only 5 samples from animals at the early pre-symptomatic phase of the CWD incubation period were collected. Just one of these samples provided positive results in the PMCA assay (20% of accurate detection, Table 2). Further PMCA rounds did not increase the efficiency or sensitivity of this assay, as assessed by using serially diluted brain extracts from CWD infected deer or blood samples (data not shown). Other technical modifications such as the use of different antibodies for detection in western blots (e.g., 6H4, 6D11 and PRC1) provided similar outcomes, as reported previously^12^.

**Table 2:** Summary of the results obtained in a blind study of detection of CWD prions in blood from white-tailed deer coding for different polymorphic versions of the prion protein (at position 96).

Biological sample	96GG^b^	96GS	96SS
Late Pre-symptomatic WTD (B+ LN+)	47/49 95.9%	7/12 58.3%	ns^c^
Early pre-symptomatic WTD (B− LN+)	18/34 52.9%	8/14 57.1%	1/5 20.0%
Negative WTD (B− LN−)	0/10 100%	0/8 100%	0/3 100%
Overall correct diagnosis^a^	80.6%^d,e^	67.6%^f,g^	50%^h,i^

^a^Samples were declared positive if at least one of the two replicates performed provided a protease-resistant PrP^Sc^ signal in the PMCA assay.

^b^Data from Kramm et al.^12^.

^c^*ns* no samples.

^d^Estimates of sensitivity for the PrP 96GG group, with 95% confidence intervals: 67.9–86.8%.

^e^Estimates of specificity for the PrP 96GG group, with 95% confidence intervals: 69.2–100%.

^f^Estimates of sensitivity for the PrP 96GS group, with 95% confidence intervals: 29.9–70.1%.

^g^Estimates of specificity for the PrP 96GS group, with 95% confidence intervals: 63.1–100%.

^h^Estimates of sensitivity for the PrP 96SS group, with 95% confidence intervals: 0.5–71.6%.

^i^Estimates of specificity for the PrP 96SS group, with 95% confidence intervals: 29.2–100%.

As mentioned, each sample was tested in four replicates. An animal was considered CWD positive by PMCA if at least one of these replicates provided positive signals. Replicates consisted of (i) direct spiking of blood in PMCA substrate, or (ii) pellets derived from the concentration of larger blood volumes (Fig. 2). From the 14 PMCA positive samples in this screening, just one of them was positive in the “spiked” version only (sample − 134, Fig. 2). This modality allowed us to assess, side by side, if the concentration of large volumes of samples is advantageous compared to direct spiking of lower volumes of the same samples in the PMCA reaction. As expected, our results suggest that sample concentration increase the diagnostic efficiency of the PMCA assay, in line with previous recommendations^10,26^.

## Discussion

CWD is problematic due to its deadly outcome and its ability to efficiently spread across wild and farmed cervid populations. Sensitive *ante-mortem* diagnostic assays would be highly useful in identifying early cases of CWD, providing a useful tool for disease management. The development of in vitro prion conversion assays (PMCA^10^ and RT-QuIC^11^) provides hope towards the development of *ante-mortem* CWD tests for easily accessible samples such as blood, feces, urine and saliva^19,27,28^. Additionally, these techniques could be utilized to detect CWD prions longitudinally in experimentally infected WTD using the above mentioned sample types. Blood is an ideal sample type for several reasons: (i) it provides the lowest chances of cross-contamination due to the aseptic nature of collection, (ii) it is routinely taken for other testing on farmed animals, and (iii) it is known to contain seeding competent prions in experimental and natural conditions^7,12,29,30^.

Previous reports show that prion-seeding activity and PrP deposition can be identified in a variety of biological samples from experimentally infected WTD shortly after infection^19,28,31^. Our previously published data^12^ show that we can detect CWD prions, to various degrees, in blood of both early and late pre-symptomatic WTD. That report focused on WTD that were G homozygous at *PRNP* codon 96^20^. We described a mean correct diagnostic identification of 80.6% in animals known to be CWD positive by IHC, including 100% specificity, 95.9% sensitivity in late pre-symptomatic animals, and 52.9% in early pre-symptomatic animals^12^. However, WTD have different *PRNP* polymorphisms at codon 96, where GG is considered to be the wild type, and GS and SS animals are less common^20^. Several reports suggest that presence of S at position 96 can modulate susceptibility to prion infection^17,18,20^, as well as peripheral replication and shedding of infectious particles^19,20^. In that sense, polymorphic variations in the prion protein may greatly affect blood-based diagnosis of field samples.

Here, we report diagnostic data using blood from a small cohort of *PRNP* 96S homozygous and heterozygous WTD. First, we tested whether our WTD PMCA (utilizing PrP 96G susbtrate) was able to replicate CWD prions from animals carrying at least one 96S allele. Our results (Fig. 1) shows that this is possible. Surprisingly, our specific WTD PMCA assay replicated brain-derived prions from the terminally ill PrP 96GS animals more efficiently compared to the ones obtained from the brain of a PrP 96GG WTD. Importantly, both sample sources were normalized for their PK-resistance PrP levels before testing them in PMCA. Whether the efficient in vitro prion replication efficiency observed here for PrP 96GS prions is reproducible is part of ongoing studies. One explanation for this outcome could be found in a potential dissimilar proportion of seeding competent prion aggregates compared to the PK-resistant signal they provide. An alternative explanation is that these samples comprise different prion strains with particular (and different) rates of PMCA conversion. Regardless, these results suggest that our PMCA system is suitable for the screening of biological samples derived from WTD carrying PrP 96S alleles.

Considering the results described above, we tested blood samples derived from WTD carrying one or two PrP 96S alleles for their presence of seeding competent CWD prions. The overall diagnostic power for this new WTD cohort substantially decreased when compared to a similar population of animals homozygous for the PrP 96G haplotype^12^. When comparing data of PrP 96GG and PrP 96GS animals, similar sensitivity was observed for both animal groups at the early pre-symptomatic stage (57.1% versus 52.9%, respectively). Unfortunately, accurate diagnosis for the late pre-symptomatic PrP 96GS deer reached a similar level of accuracy (58.3%) when compared to subjects at the early pre-symptomatic phase. These values contrasted with the ones found for the PrP 96GG cohort where correct diagnosis at late pre-symptomatic stages reached 95.9%^12^. If we consider that just 1 out of 5 (20%) of the CWD-infected PrP 96SS deer was accurately diagnosed, we can assume that the PrP 96S polymorphism modulates the presence of CWD prions in peripheral compartments, such as blood, in a dose dependent manner, especially at late stages of the CWD incubation periods. In addition, our data suggest that the presence of CWD prions in peripheral compartments reach their intrinsic maximum levels early during the incubation periods when the PrP 96S allele is present. Future research using controlled experimental conditions may confirm or refute the previously mentioned assumption.

Similarly as described in our previous report^12^, some of the tested samples did not result in positive PMCA detection all the times they were tested. In that sense, our current protocol require several replicates in order to provide accurate results. Additional tests assessing for the reproducibility of our assays were not possible due to the limited amount of sample available. Nevertheless, several optimization studies are currently ongoing in our laboratory using other blood sources and biological specimens. One explanation for the lower detection efficiency in samples from deer carrying at least one *PRNP* 96S allele may be found in potential incompatibilities between the PrP^Sc^ CWD inocula and PMCA substrates. Compelling evidence in several animal species, including WTD, show that polymorphic variation in the prion protein may modulate the susceptibility of disease transmission^18,32–34^. Although the use of PrP 96GG PMCA substrate may have had an effect on our results, the data presented in Fig. 1 suggests that this specific protocol can effectively replicate prions from both PrP 96GS and PrP 96GG origins equally. Unfortunately, PrP 96SS substrate was not available in our laboratory at the moment of running this experiment, and blood samples from WTD available to us were restricted to what was used in this study. This limited further studies using homologous substrate for blood samples derived from PrP 96SS WTD. In addition, we do not know the distribution of each prion allele with respect to the number of PrP^Sc^ particles present in brain and blood of infected animals. In that line, the concentration of infectious prion proteins present in peripheral versus brain compartments may be different between PrP 96GG and PrP 96GS animals and could have contributed to the lower diagnostic sensitivity observed in this study. If we additionally consider the possibility that these animals are naturally infected with different strains of the CWD agent (each one of them with specific tropisms to peripheral tissues and PMCA replication properties) the scenario becomes even more complex. Studies in these directions may help to not only further our understanding on natural CWD transmission, but also contribute in refining diagnostic methods.

Our results suggest that blood detection using the current PMCA protocol is limited, especially in early stages of the CWD incubation period. Nevertheless, practices like repeated sampling at different times and improvements in the PMCA protocol (at the sample processing or the prion replication stages), enrichment of certain blood components (e.g., buffy coat), testing PrP polymorphic -specific PMCA substrates, among others, may improve the numbers presented in this article. Another limitation of this study involves the limited sample size interrogated by PMCA. This is more pronounced for the PrP 96 SS samples as just 5 specimens comprising only the early pre-symptomatic stage were tested. In that sense, we acknowledge that further studies utilizing larger cohorts from all polymorphic groups and using complementary techniques (e.g., RT-QuIC) are needed to confirm our findings. The evaluation of CWD-prions content in different blood fractions^29^ for different polymorphic groups may also help not only to improve detection efficiency, but also to provide information on peripheral prion replication for WTD harboring different PrP polymorphic variants. In addition, bioassays would further confirm whether prion infectivity in blood is correlated with in vitro prion detection. Nevertheless, this data suggest that the *PRNP* codon 96 polymorphisms in WTD influence the ability to detect CWD in blood samples and needs to be considered in advance when pursuing *ante-mortem* diagnostic tests.

## Supplementary information

Supplementary Information.
