# Supplementary material for: Chronic wasting disease (CWD) prion detection in blood from pre-symptomatic white-tailed deer harboring PRNP polymorphic variants
Source: Sci Rep. 2020 Nov 13;10:19763. doi: 10.1038/s41598-020-75681-7 (PMC7666123; doi:10.1038/s41598-020-75681-7)
Supplement: Supplementary file 1 — Supplementary Information. [file 41598_2020_75681_MOESM1_ESM.pdf]

SUPPLEMENTAL FILES

Chronic wasting disease (CWD) prion detection in blood from pre-symptomatic white-tailed deer harboring *PRNP* polymorphic variants.

Carlos Kramm[1,2], Paulina Soto[1], Tracy A. Nichols[3] and Rodrigo Morales[1,4*].

[1]Department of Neurology. McGovern Medical School. The University of Texas Health Science Center at Houston. Houston, TX 77030, USA.

[2]Universidad de Los Andes. Facultad de Medicina, Av. San Carlos de Apoquindo 2200. Las Condes, Santiago, Chile.

[3]United States Department of Agriculture, Animal Plant Health Inspection Service, Veterinary Services, Fort Collins, CO 80526, USA.

[4]CIBQA, Universidad Bernardo O'Higgins. Santiago, Chile.

* To whom correspondence should be addressed: Rodrigo.MoralesLoyola@uth.tmc.edu

Figure 1

A)

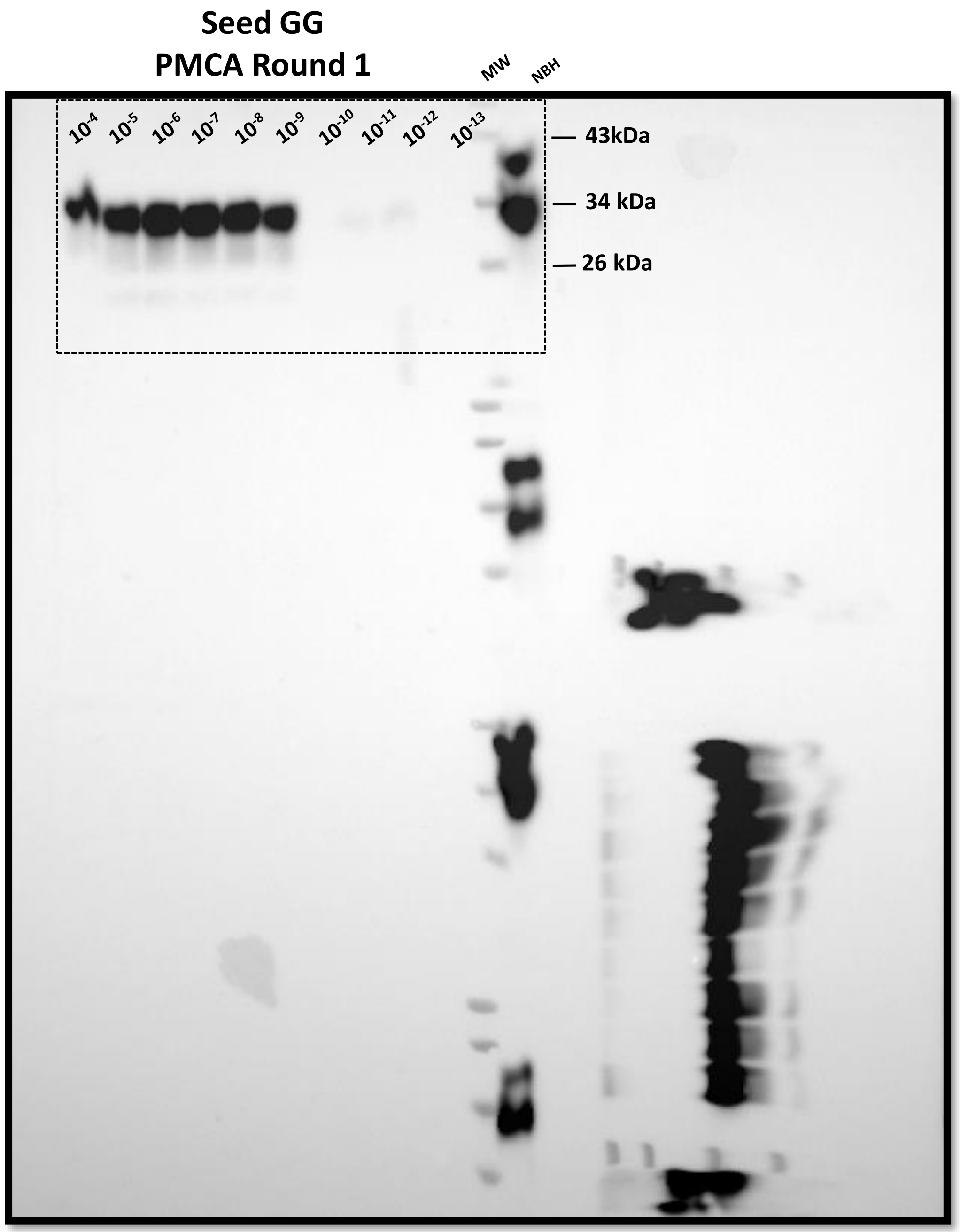
Seed GG
PMCA Round 1
MW
NBH
10-4
10-5
10-6
10-7
10-8
10-9
10-10
10-11
10-12
10-13
43kDa
34 kDa
26 kDa

Figure 1

A)

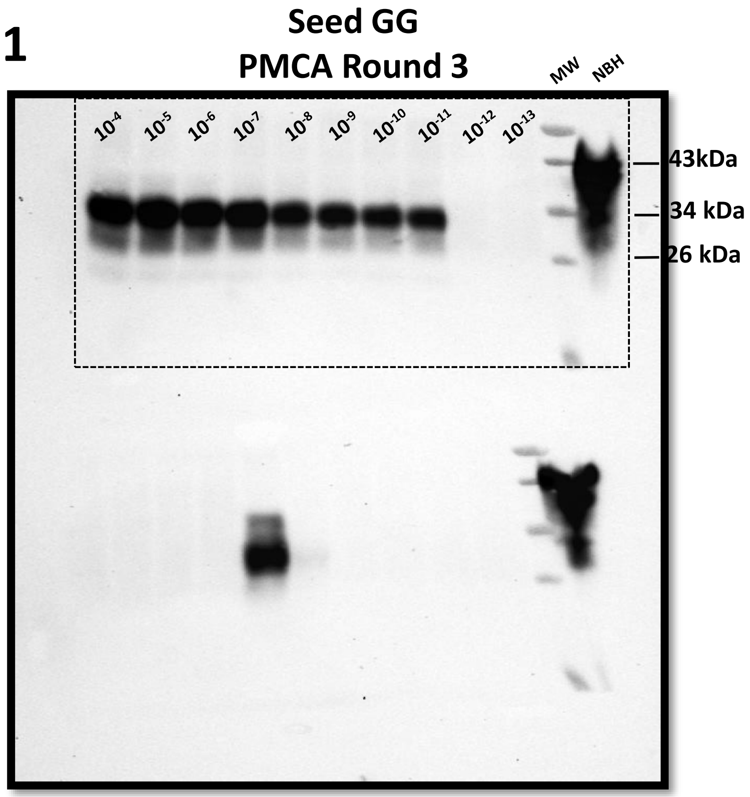
Seed GG
PMCA Round 3
MW
NBH
10^{-4}
10^{-5}
10^{-6}
10^{-7}
10^{-8}
10^{-9}
10^{-10}
10^{-11}
10^{-12}
10^{-13}
43kDa
34 kDa
26 kDa

Figure 1

B)

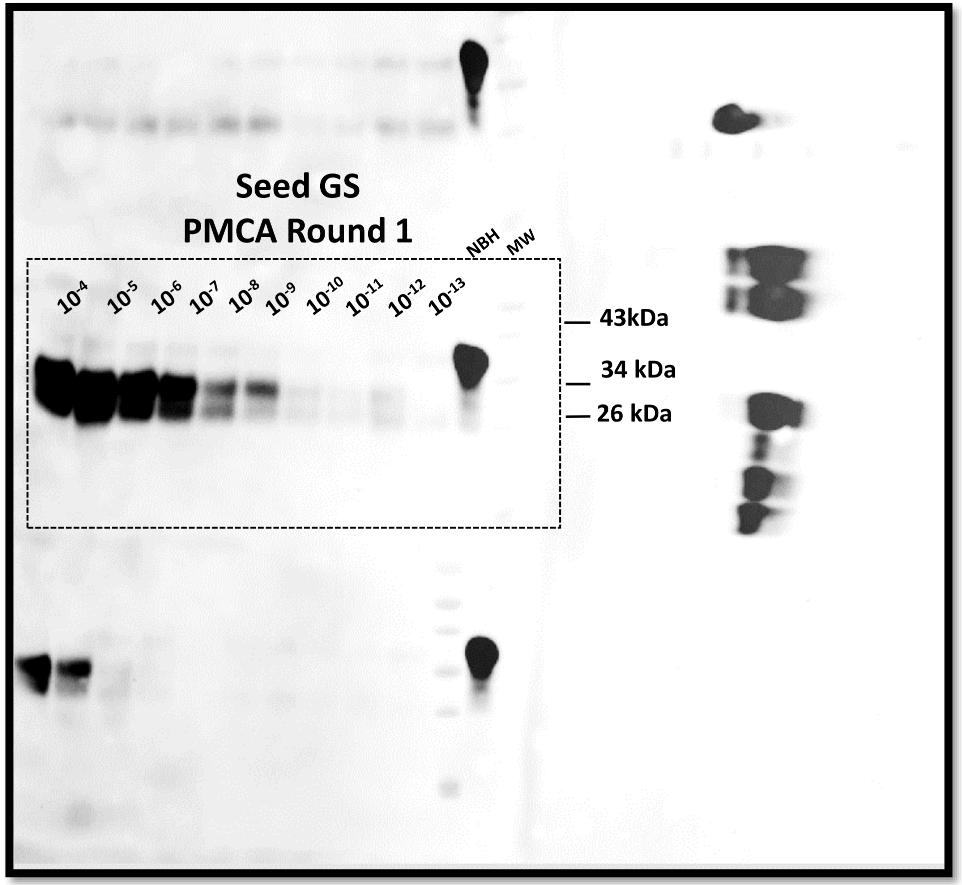
Seed GS
PMCA Round 1
NBH
MW
10-4
10-5
10-6
10-7
10-8
10-9
10-10
10-11
10-12
10-13
43kDa
34 kDa
26 kDa

Figure 1

B)

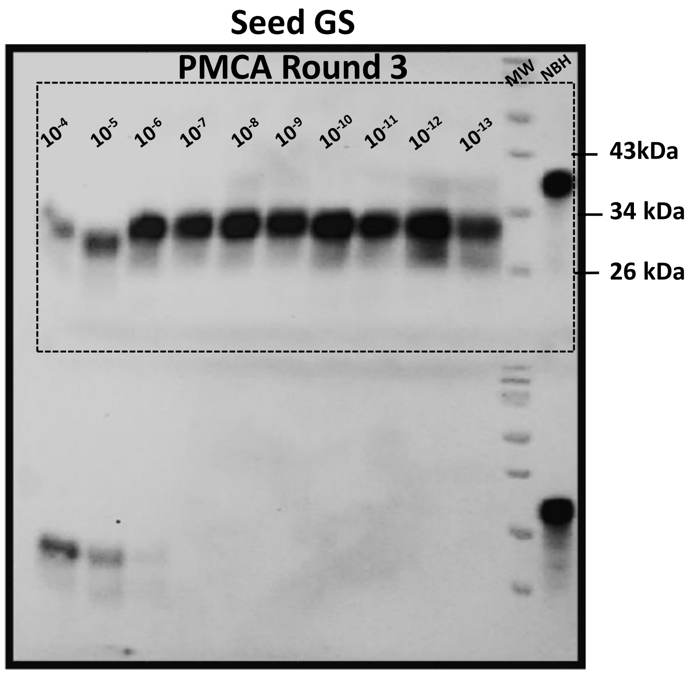
Seed GS
PMCA Round 3
MW
NBH
10^{-4}
10^{-5}
10^{-6}
10^{-7}
10^{-8}
10^{-9}
10^{-10}
10^{-11}
10^{-12}
10^{-13}
43kDa
34 kDa
26 kDa

Figure 2

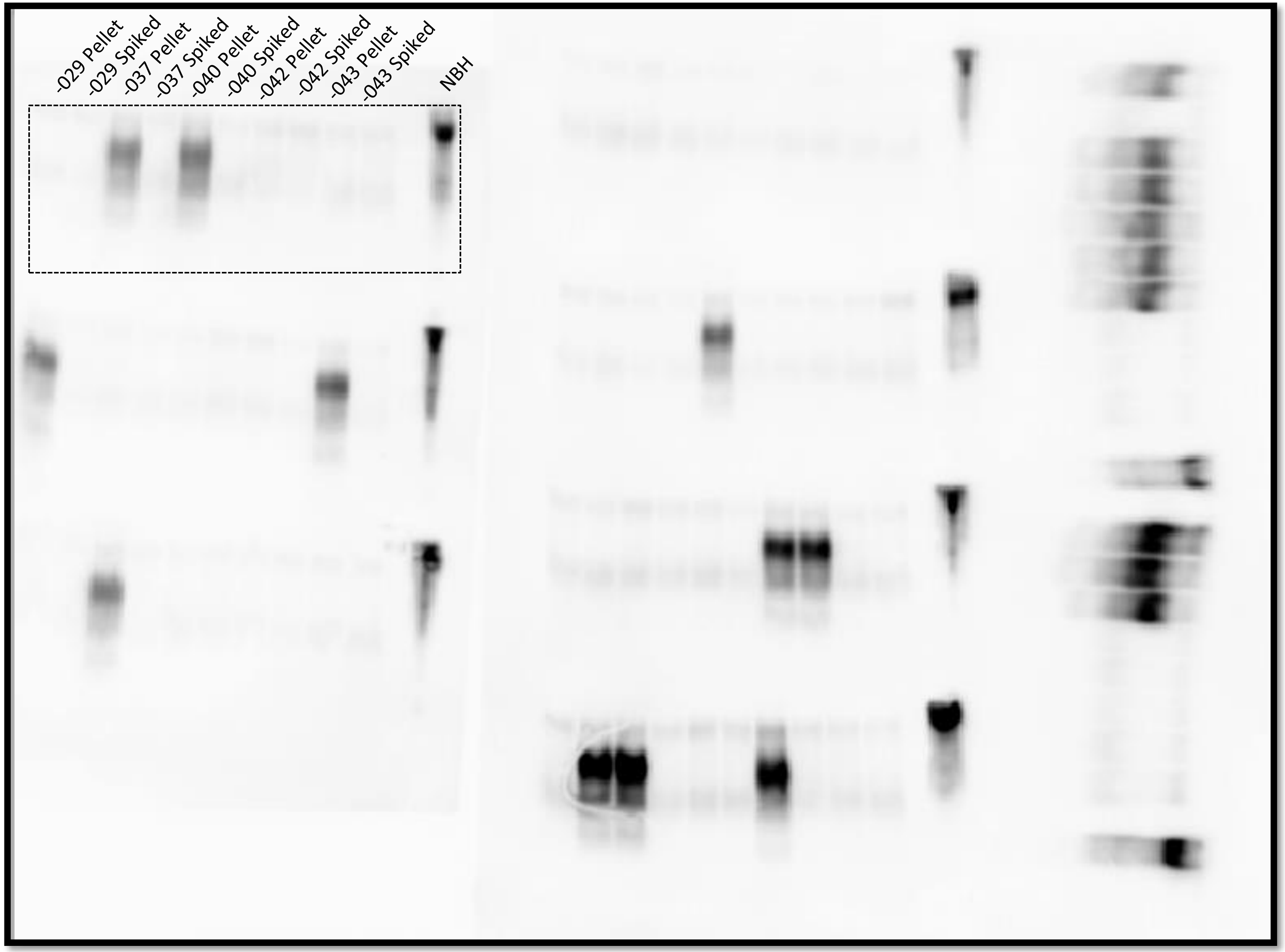
-029 Pellet
-029 Spiked
-037 Pellet
-037 Spiked
-040 Pellet
-040 Spiked
-042 Pellet
-042 Spiked
-043 Pellet
-043 Spiked
NBH

Figure 2

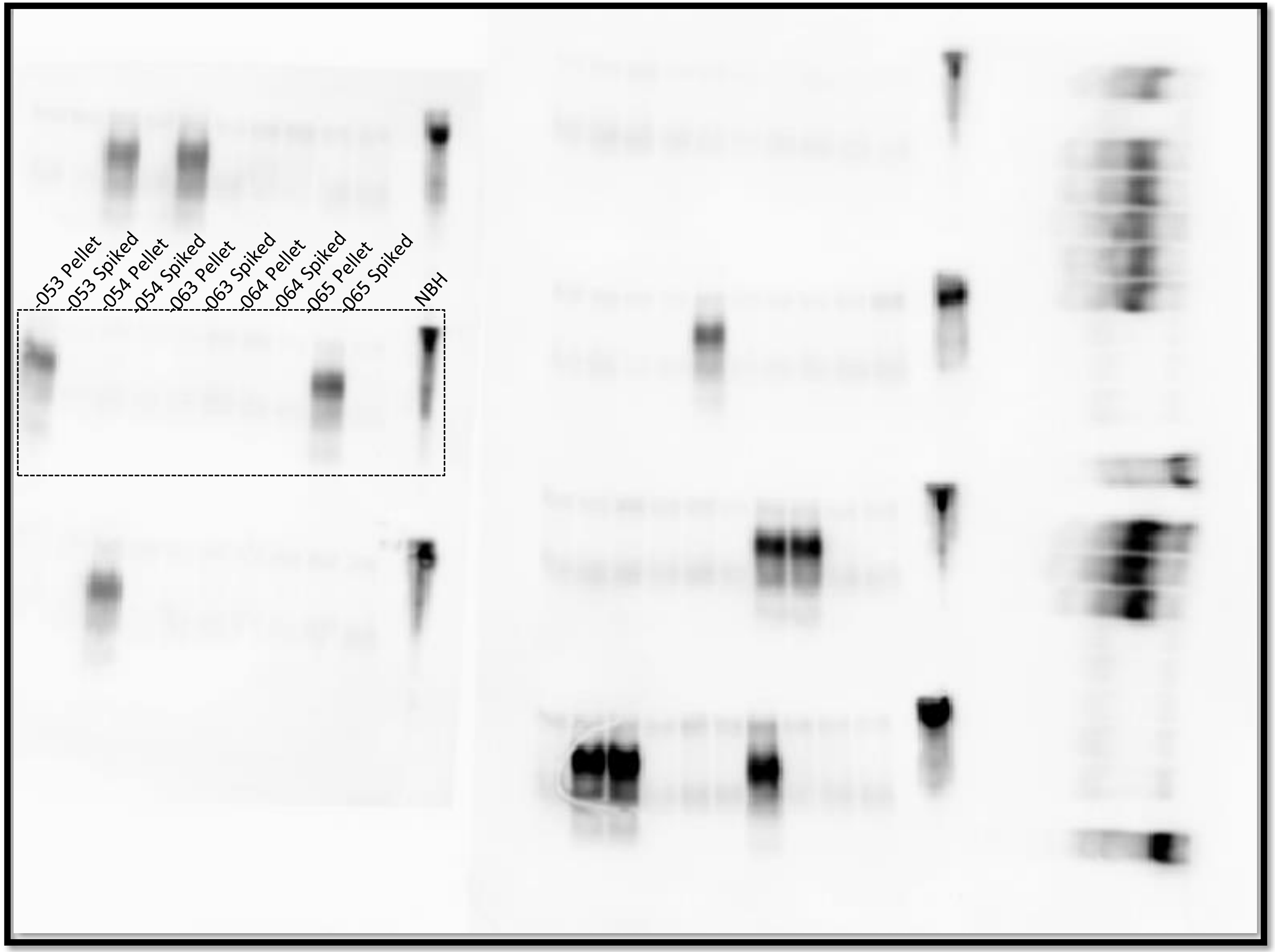

-053 Pellet
-053 Spiked
-054 Pellet
-054 Spiked
-063 Pellet
-063 Spiked
-064 Pellet
-064 Spiked
-065 Pellet
-065 Spiked
NBH

Figure 2

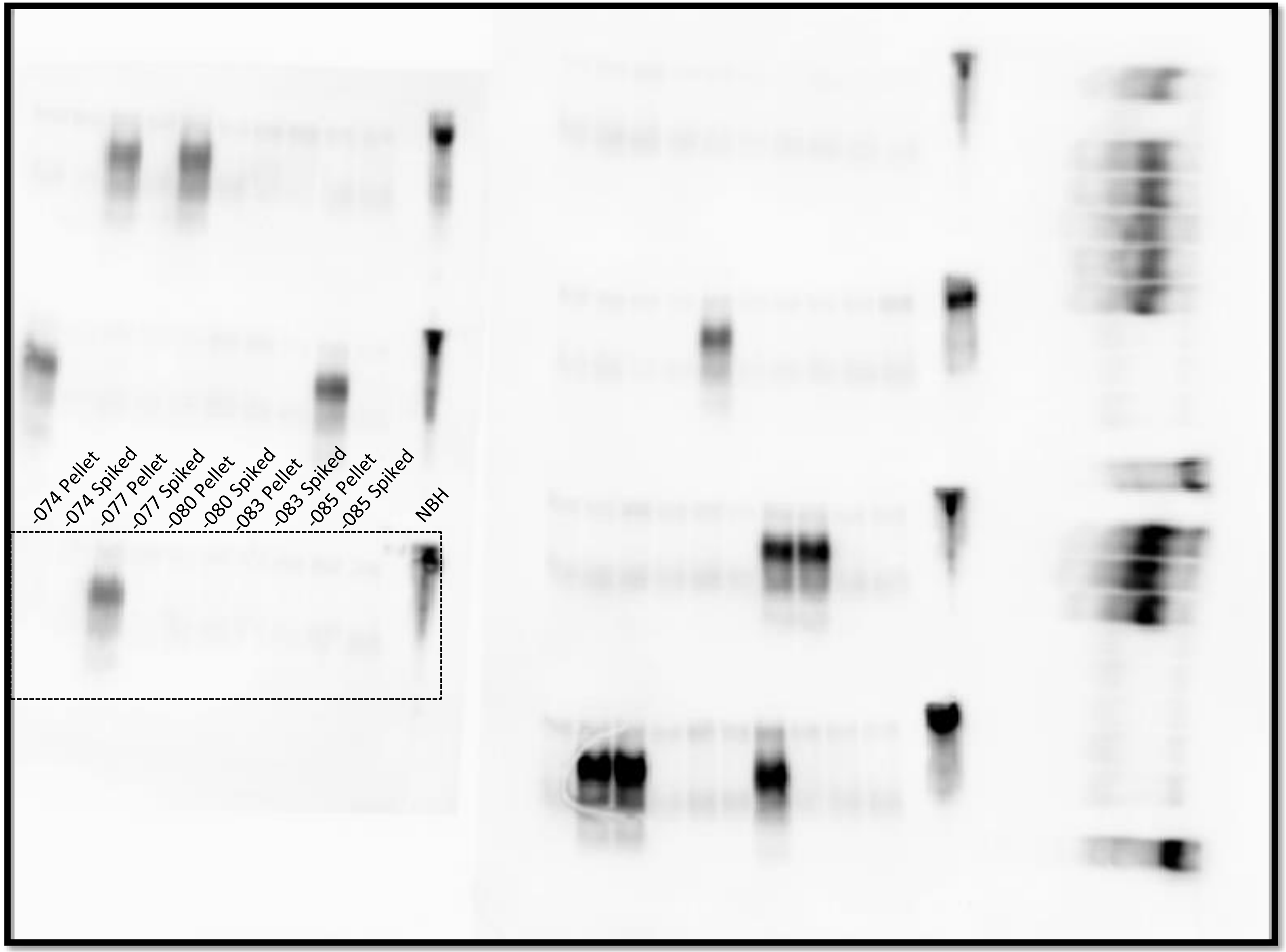
-074 Pellet
-074 Spiked
-077 Pellet
-077 Spiked
-080 Pellet
-080 Spiked
-083 Pellet
-083 Spiked
-085 Pellet
-085 Spiked
NBH

Figure 2

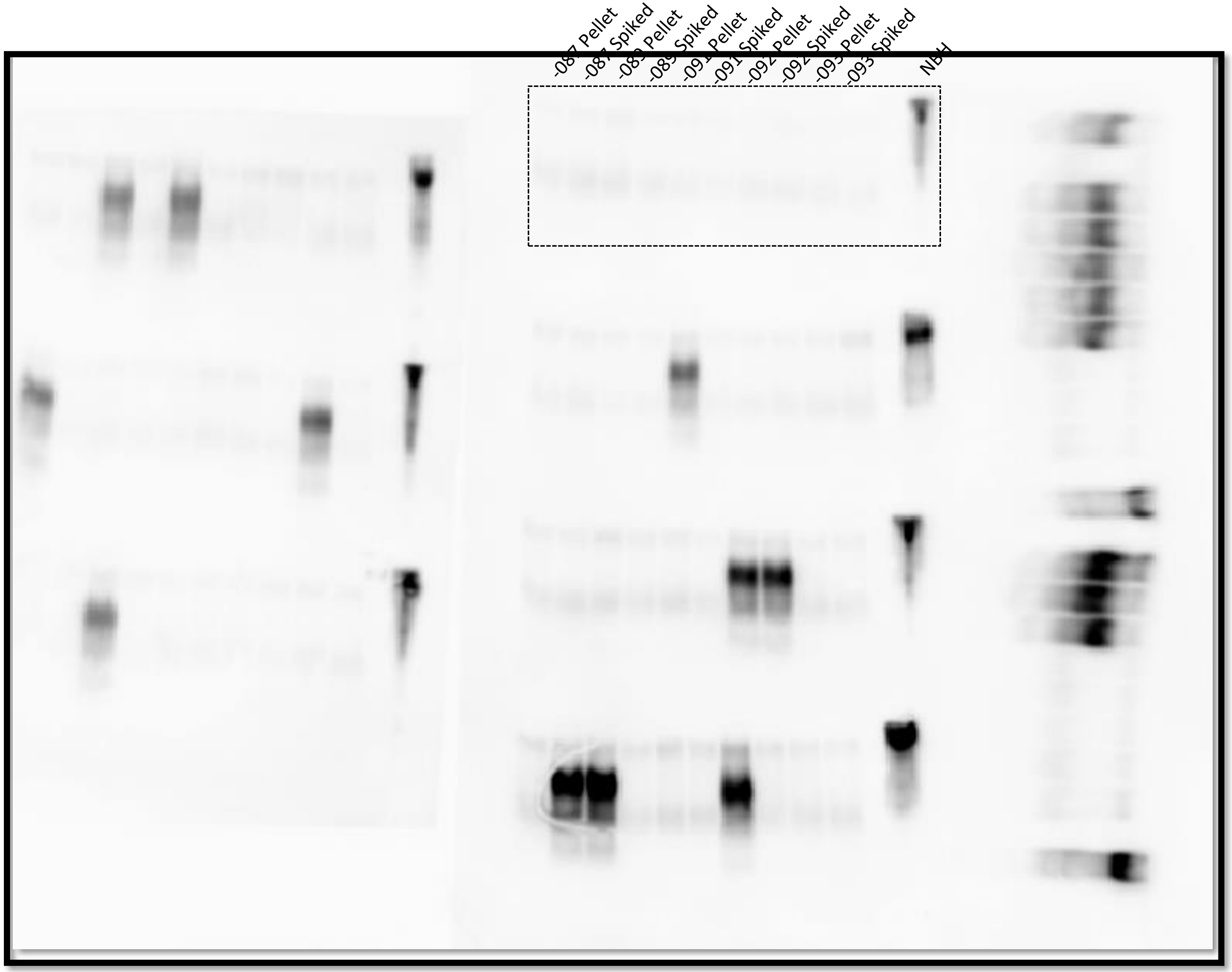
-087 Pellet
-087 Spiked
-089 Pellet
-089 Spiked
-091 Pellet
-091 Spiked
-092 Pellet
-092 Spiked
-093 Pellet
-093 Spiked
NBH

Figure 2

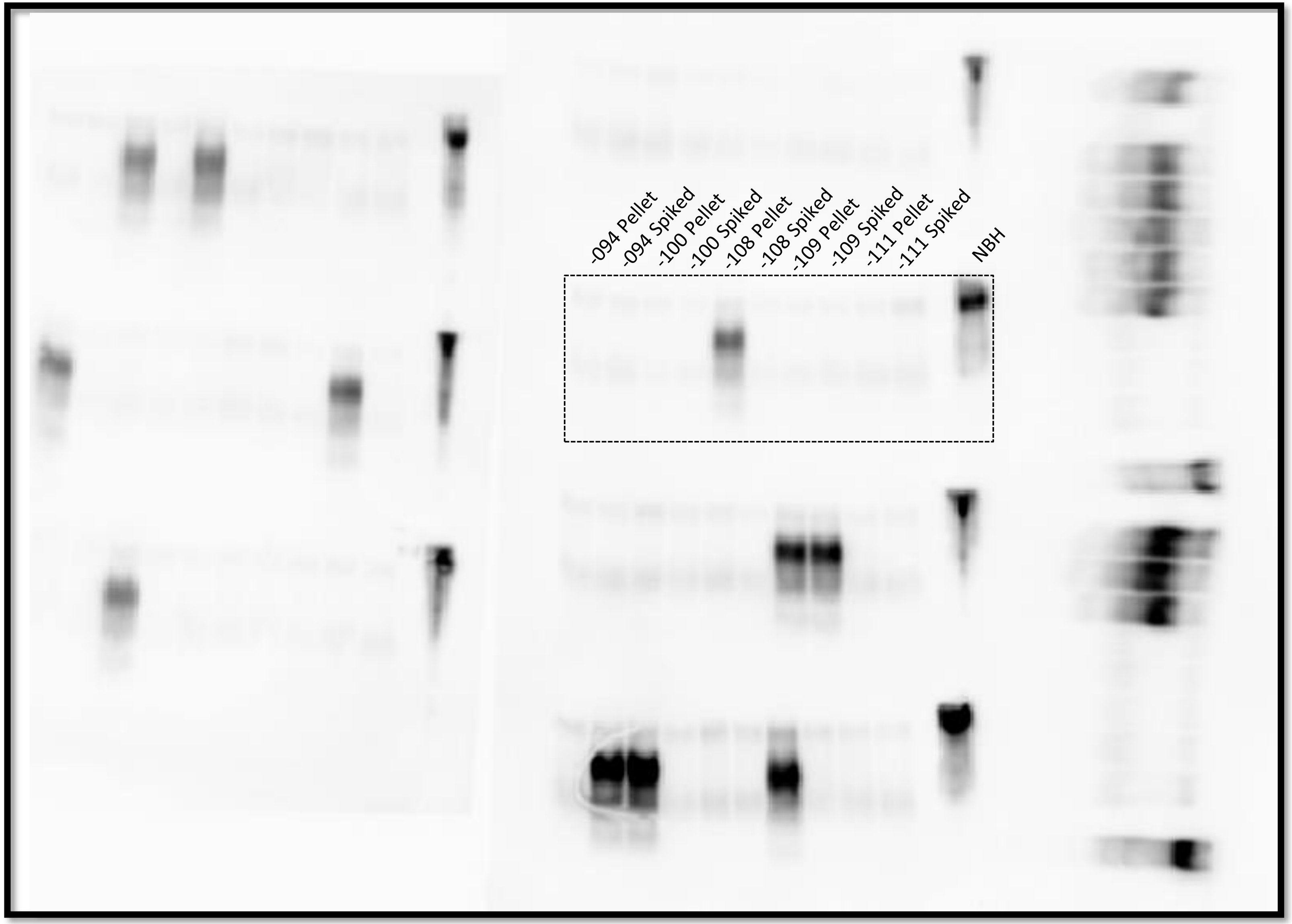
-094 Pellet
-094 Spiked
-100 Pellet
-100 Spiked
-108 Pellet
-108 Spiked
-109 Pellet
-109 Spiked
-111 Pellet
-111 Spiked
NBH

Figure 2

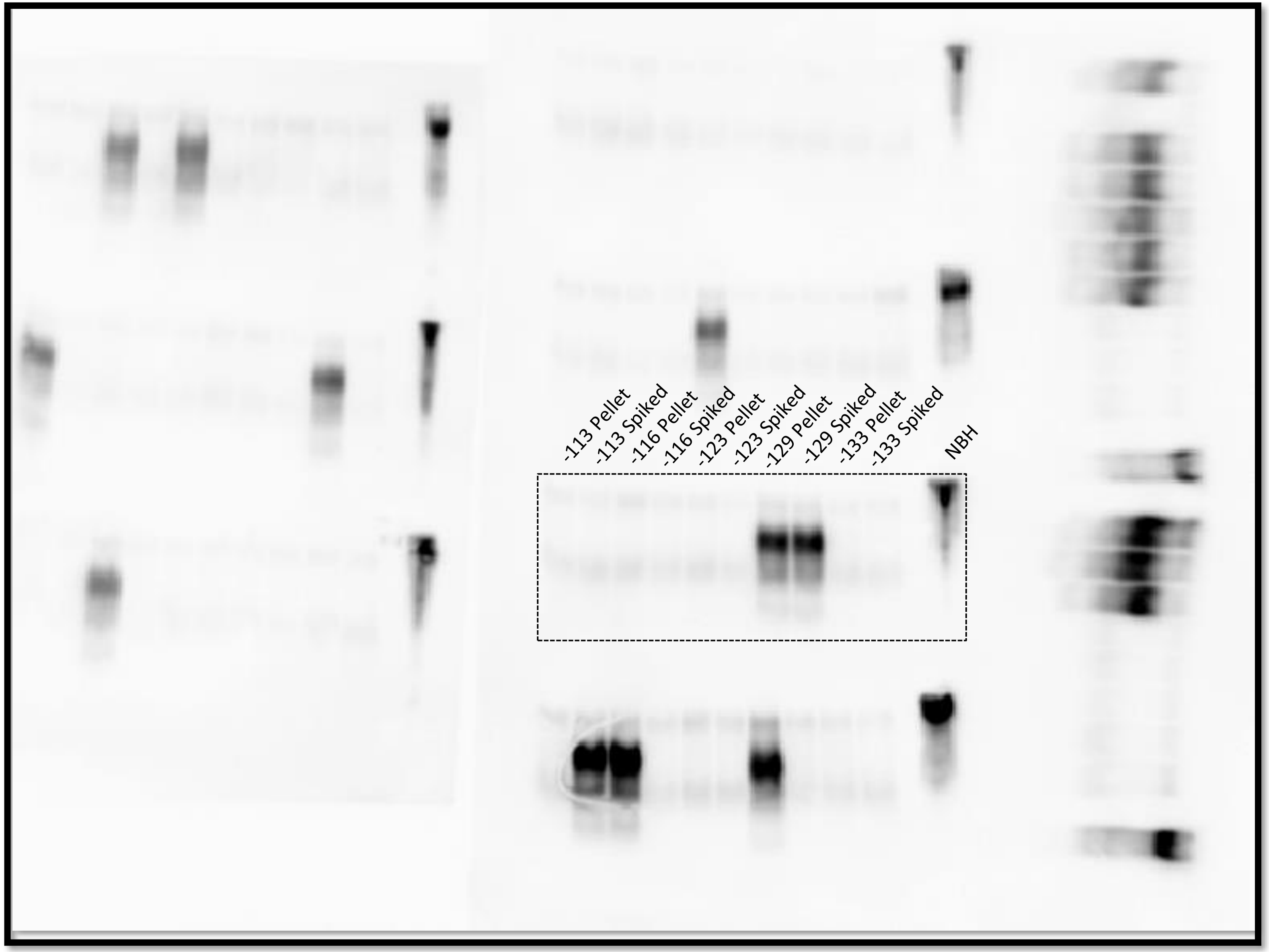
-113 Pellet
-113 Spiked
-116 Pellet
-116 Spiked
-123 Pellet
-123 Spiked
-129 Pellet
-129 Spiked
-133 Pellet
-133 Spiked
NBH

Figure 2

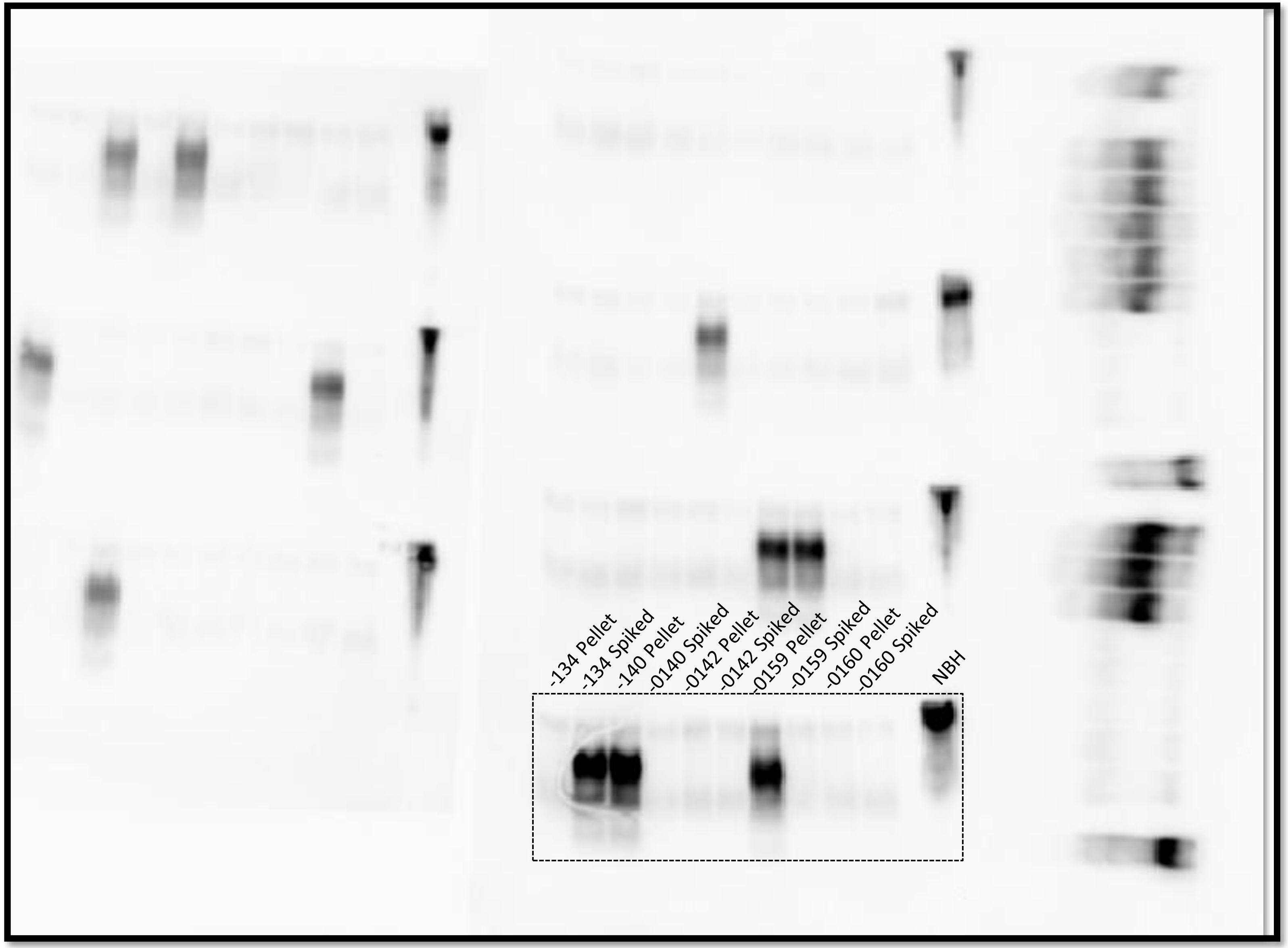
-134 Pellet
-134 Spiked
-140 Pellet
-0140 Spiked
-0142 Pellet
-0142 Spiked
-0159 Pellet
-0159 Spiked
-0160 Pellet
-0160 Spiked
NBH

Figure 2

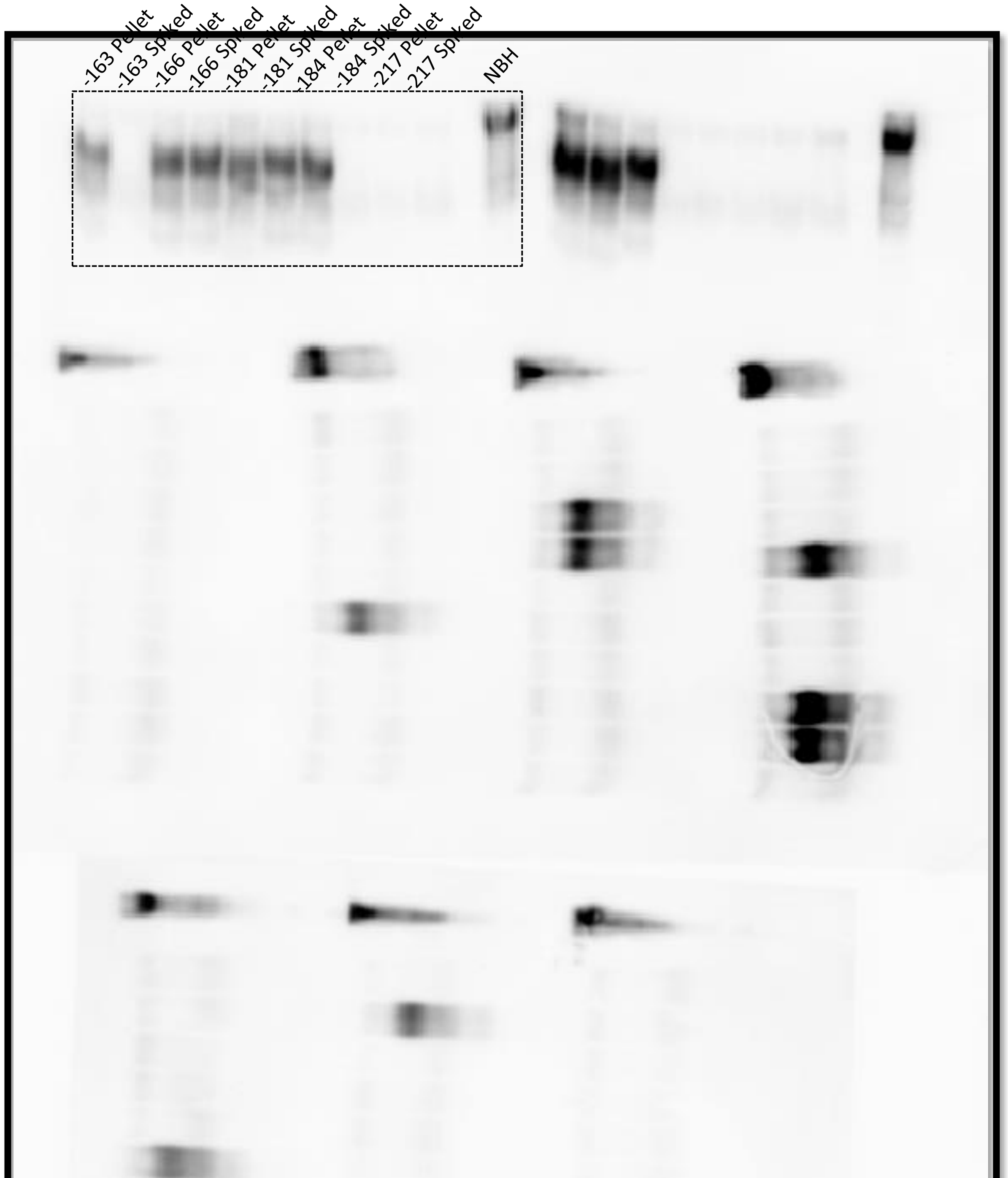
-163 Pellet
-163 Spiked
-166 Pellet
-166 Spiked
-181 Pellet
-181 Spiked
-184 Pellet
-184 Spiked
-217 Pellet
-217 Spiked
NBH

Figure 2

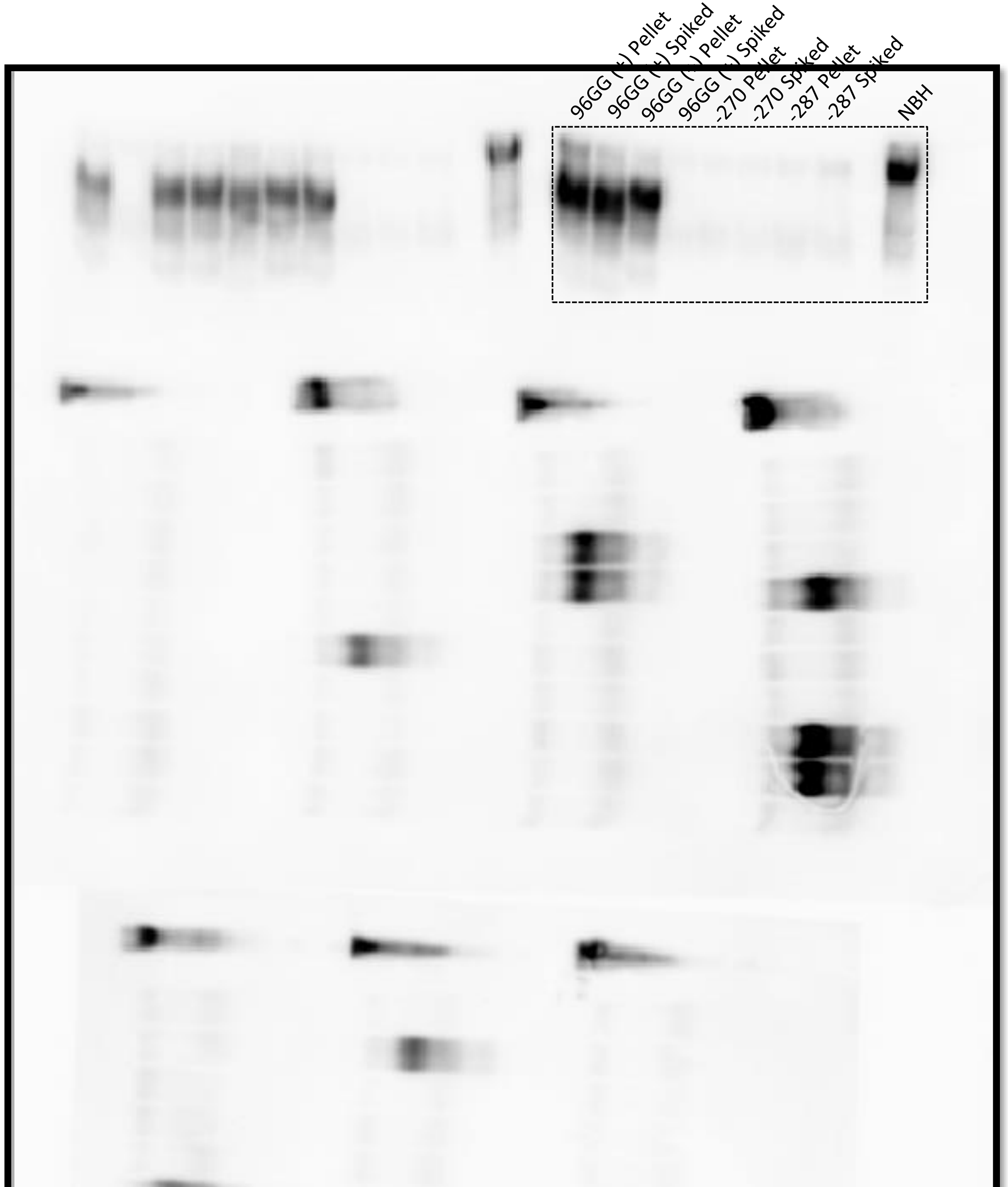

96GG (+) Pellet
96GG (+) Spiked
96GG (-) Pellet
96GG (-) Spiked
-270 Pellet
-270 Spiked
-287 Pellet
-287 Spiked
NBH
